# Processing of Odor Information During the Respiratory Cycle in Mice

**DOI:** 10.3389/fncir.2022.861800

**Published:** 2022-03-31

**Authors:** Kensaku Mori, Hitoshi Sakano

**Affiliations:** ^1^RIKEN Center for Brain Science, WAKO, Saitama, Japan; ^2^Department of Brain Function, School of Medical Sciences, University of Fukui, Fukui, Japan

**Keywords:** olfactory circuitry, odor perception, respiratory cycle, innate/learned decisions, retronasal/orthonasal odors

## Abstract

In the mouse olfactory system, odor signals detected in the olfactory epithelium are converted to a topographic map of activated glomeruli in the olfactory bulb. The map information is then conveyed by projection neurons, mitral cells and tufted cells, to various areas in the olfactory cortex. An odor map is transmitted to the anterior olfactory nucleus by tufted cells for odor identification and recollection of associated memory for learned decisions. For instinct decisions, odor information is directly transmitted to the valence regions in the amygdala by specific subsets of mitral cells. Transmission of orthonasal odor signals through these two distinct pathways, innate and learned, are closely related with exhalation and inhalation, respectively. Furthermore, the retronasal/interoceptive and orthonasal/exteroceptive signals are differentially processed during the respiratory cycle, suggesting that these signals are processed in separate areas of the olfactory bulb and olfactory cortex. In this review article, the recent progress is summarized for our understanding of the olfactory circuitry and processing of odor signals during respiration.

## Introduction

In mammals, the olfactory system plays an important role in searching for food, avoiding danger, and finding partners for survival of the individuals and species. Odor information is detected by odorant receptor (OR) molecules expressed by olfactory sensory neurons (OSNs) in the olfactory epithelium (OE) (Buck and Axel, 1991). In each OSN, only one functional OR gene is expressed in a monoallelic manner and OSN axons expressing the same OR species converge to specific target sites in the OB forming glomerular structures (Sakano, 2010). Thus, binding signals of odorants are converted to map information of activated glomeruli (Mori and Sakano, 2011). The OB is not simply a projection screen to generate odor maps, but is also composed of various functional domains for innate odor qualities (Kobayakawa et al., 2007). The map information is then conveyed by tufted cells (TCs) to the anterior olfactory nucleus (AON) to identify the input odor and to recollect the associated memory of odor experience (Igarashi et al., 2012; Aqrabawi and Kim, 2018). The recalled odor scene further activates the valence network in the amygdala that was previously connected to the memory engram for learned decisions (Mori and Sakano, 2021).

**FIGURE 1:**
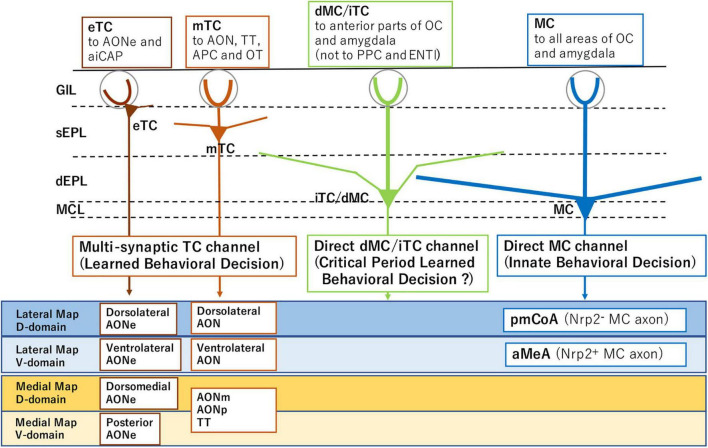
Subsets of projection neurons for distinct behavioral decisions. External tufted cells (eTC) and middle tufted cells (mTC) serve as multi-synaptic channels to the amygdala for learned behavioral decisions. Mitral cells (MC) are direct channels to induce innate behavioral decisions. Internal tufted cells (iTC) and displaced mitral cells (dMC) may serve as direct channels for imprinted behavioral decisions. The diagram also illustrates distinct projection targets of MCs and TCs in each domain of the olfactory bulb (OB). Examples of domain-specific targets are shown for MCs and TCs in the lower boxes. Abbreviations: aiCAP, the most anterolateral isolation of the cap compartment of the olfactory tubercle (OT); aMeA, anterior region of the medial amygdala; AON, anterior olfactory nucleus; AONe, AON external part; AONm, AON medial part; AONp, AON posterior part; APC, anterior piriform cortex; ENTl, lateral entorhinal area; pmCoA, posteromedial cortical amygdala; PPC, posterior piriform cortex; TT, tenia tecta; Nrp2, Neuropilin 2; D, dorsal; and V, ventral.

Separately from the memory-based learned decisions, odor signals from a specific functional domain of the OB are directly transmitted by mitral cells (MCs) to valence regions in the amygdala to elicit innate odor responses (Figure 1; Miyamichi et al., 2011; Root et al., 2014; Inokuchi et al., 2017). Although the basic architecture of hard-wired circuits is generated according to a genetic program, stereotyped innate decisions can be modified by olfactory experiences during the critical period. Neonatal odor exposure to environmental odors affects odor perception later in life. This odor-induced imprinting imposes the positive quality on imprinted memory, even when the odor quality is innately aversive (Inoue et al., 2021).

Olfactory perception takes place in relation to the respiratory cycle (Kepecs et al., 2006; Mori and Manabe, 2014). An interesting feature of olfaction is that the system receives the orthonasal/exteroceptive information during inhalation isolated from the environmental odor world in the exhalation phase. In contrast, the system detects the retronasal/interoceptive information during exhalation isolated from the internal odor world in the inhalation phase. As each respiration cycle is composed of inhalation and exhalation, sampling of external and internal odor information is discontinuous and discrete.

In response to the orthonasal signals, MCs and TCs are separately activated during respiration in the direct and multi-synaptic pathways, respectively (Mori and Sakano, 2021). In addition, external/exteroceptive and internal/interoceptive odor signals are sequentially processed during the respiratory cycle using both MCs and TCs. Thus, each odor input is differentially processed in separate respiratory phases by distinct sets of projection neurons. In this review article, we will provide an overview of the recent progress made in understanding the olfactory circuitry and odor detection during the respiratory cycle in mice.

### Olfactory Circuitry and Behavioral Decisions

In mice, olfactory map information in the OB is transmitted by TCs to the AON for odor identification and recollection of associated scene. The recalled memory-engram further activates the valence network that was connected in the previous odor experience (Mori and Sakano, 2021). In contrast, for innate behavioral decisions, olfactory information is directly conveyed by distinct subsets of MCs to specific valence regions in the amygdala (Inokuchi et al., 2017).

#### Direct Pathway for Innate Olfactory Decisions

During the process of OSN projection to the OB, olfactory information is roughly sorted into two distinct qualities, aversive and attractive along the dorsal/ventral (D/V) axis of the OB (Inokuchi et al., 2017). A pair of signaling molecules, Neuropilin 2 (Nrp2) and its repulsive ligand Semaphorin 3F (Sema3F) are involved in this segregation. The Nrp2^–^/Sema3F^+^ OSNs project their axons to the dorsal (aversive/fear) domain in the OB, whereas the Nrp2^+^/Sema3F^–^ OSNs project to the postero-ventral (attractive) domain (Takeuchi et al., 2010).

To elicit the instinct behavioral responses, projection neuron MCs are subdivided into at least two subsets based on the expression of an axon guidance molecule, Nrp2. The Nrp2^–^ MCs transmit the negative-valenced signals, whereas the Nrp2^+^ MCs convey the positive-valenced orthonasal/exteroceptive signals to the amygdala (Inokuchi et al., 2017).

During development, MC precursors are born in the ventricular zone in the embryonic OB and differentiate into two separate subsets, Nrp2^+^ and Nrp2^–^. After migrating radially to the surface of the embryonic OB, Nrp2^+^ MCs further migrate tangentially down to the ventral side directed by Sema3F secreted from the dorsal-zone OSN axons (Inokuchi et al., 2017). Common usage of the same Nrp2/Sema3F signaling for both OSN projection and MC migration is important to transmit the olfactory information correctly within the glomeruli. This is because matching of the OSN axons and MC dendrites takes place based on the proximity between one another (Nishizumi et al., 2019).

Loss-of-function and gain-of-function experiments revealed that activation of a single axon guidance gene *Nrp2* is sufficient to initiate the circuit formation of two separate pathways, aversive (Nrp2^–^) and attractive (Nrp2^+^), each connecting to the posteromedial cortical amygdala (pmCoA) and the anterior region of the medial amygdala (aMeA), respectively (Inokuchi et al., 2017; Figure 1). It is also shown that MC-specific knockout (KO) of the *Nrp2* abolishes ultrasonic vocalization of male mice induced by female scents. Furthermore, forced expression of human Nrp2 in the dorsal-lineage MCs causes rerouting of their axons to the aMeA (Inokuchi et al., 2017).

#### Interpretation of Olfactory Map for Behavioral Decisions

As already mentioned, odor signals detected by ORs in the OE are converted to a topographic information. However, the glomerular map not only serves as a projection screen for odor maps but also contains distinct functional domains for innate odor qualities (Mori and Sakano, 2011). For odor identification and discrimination, map information is transmitted to the AON by TCs for learned decisions. In contrast, for instinct decisions, it was not clear whether the combinatorial pattern of activated glomeruli is utilized as a whole, or if each individual glomeruli possess a specialized function. Using an optogenetic method, it was shown that photo-activation of a single glomerular species responsive to a fox odor, trimethyl thiazoline (TMT), induces freezing but not stress-induced aversion mediated by adrenocorticotropic hormone (ACTH) (Saito et al., 2017). These observations indicate that single glomerular species can elicit a particular behavioral response and that TMT-induced fear can be divided into two separate components, immobility and avoidance.

### Imprinted Memory Can Change the Innate Odor Quality

The odor quality is innately determined by the hard-wired pathway, such as for attractive smells of fruits and aversive scents of spoiled food. For learned olfactory decisions, odor perception is affected by the scene’s memory associated with the previous odor experience. Moreover, environmental odor exposure in neonates causes an irreversible change of odor quality. This phenomenon known as imprinting imposes the positive quality on imprinted memory, even when the odor quality is innately aversive, e.g., 4-methylthiazol (4MT) (Inoue et al., 2021).

#### Sema7A Signaling Is Needed for Olfactory Imprinting During the Critical Period

Imprinting was discovered in the bird visual system by Lorenz (1935). Ducklings follow the first moving object that they see after hatching recognizing it as a parental bird. Although this phenomenon is widely known, its molecular basis had largely been unknown at the circuit level. Recently, significant progress was made in the mouse olfactory system for imprinting.

Odor experience during the critical period induces olfactory imprinting by increasing the sizes of responsive glomeruli in the neonatal OB. This enlargement causes an increase in the sensitivity to the imprinted odor. A pair of signaling molecules, Sema7A and its receptor Plexin C1 (PlxnC1), are responsible for enlarging the odor-stimulated glomeruli by inducing the post-synaptic events and primary-dendrite selection (Inoue et al., 2018). Sema7A is expressed at the axon termini of OSNs in an activity-dependent manner, whereas PlxnC1 is localized to the M/T-cell dendrites only during the first week after birth. KO studies and naris-occlusion experiments revealed that blocking of Sema7A/PlxnC1 signaling during the critical period results in the impairment of smooth social interactions as adults (Inoue et al., 2021).

#### Oxytocin Is Involved in Imposing the Positive Quality on Imprinted Memory

Elevated sensitivity is a form of olfactory imprinting that is established at the level of glomeruli, and the blockage of Sema7A signaling can be explained by the impairment of odor-evoked synapse formation. However, lasting interest or positive response is another feature of olfactory imprinting. How is it that the perceptive change occurs for the experienced odors? What is responsible for establishing smooth social interactions by imprinted memory? Oxytocin in neonates appears to be involved in olfactory imprinting (Inoue et al., 2021). In the oxytocin KO, the sensitivity to the imprinted odor is increased, however, positive responses are not promoted. Intra-peritoneal injection of oxytocin into the oxytocin KO rescues the impairment of social responses when injection is given during the critical period. Imprinted odor memory reduces the stress by lowering the plasma concentration of a stress hormone, ACTH. This relaxation effect cannot be seen in the oxytocin-KO. These observations indicate that separately from Sema7A signaling, oxytocin in neonates is responsible for imposing the positive quality on imprinted memory.

### Sequential Transmission of Exteroceptive Odor Signals During Respiration

Tufted cells and MCs transmit inhalation-phased external signals at different but overlapping time windows of the respiratory cycle. The OB contains at least four distinct types of projection neurons: external TCs, middle TCs, internal TCs, and MCs (Mori, 1987; Hayar et al., 2004; Shepherd et al., 2004; Hirata et al., 2019). Recording of odor-induced firing revealed that these projection neurons convey odor signals to the different areas in the olfactory cortex (OC) and amygdala (Igarashi et al., 2012). Since odor information is separately transmitted to the OC and amygdala by different sets of projection neurons, it is interesting to study how the odor signals are differentially distributed to the central brain for innate and learned decisions during respiration.

#### Differential Activation of Innate and Learned Olfactory Circuits

During inhalation, external TCs mediate the earliest transmission of odor signals to small areas in the olfactory peduncle, showing high-frequency burst discharges beginning at the rising phase of inhalation and lasting through the end of inhalation. Middle TCs also mediate the early transmission mainly to the olfactory peduncle in the OC, demonstrating high-frequency burst discharges starting at the rising phase of inhalation and continuing until the middle part of exhalation. During inhalation, the high-frequency burst discharges of TCs generate gamma-oscillatory local-field potentials that travel to the anterior piriform cortex (APC) through the AON (Mori et al., 2013). This observation suggests that for learned behavioral decisions, the multi-synaptic TC pathway to higher areas via the AON and APC processes the external odor information during inhalation and early exhalation.

In contrast to the TC pathway, MCs convey the external odor signals mostly during exhalation, directly to widespread areas in the OC and CoA, and show low-frequency burst firing starting at early exhalation and lasting through the end of exhalation. This suggests that the direct MC pathway conveys the external odor information to the OC and amygdala during exhalation for innate behavioral decisions.

The pattern and duration of the inhalation/exhalation cycle correlate well with various behavioral states, e.g., movement state, awake resting state, and eating state (Mori and Manabe, 2014). Regardless of state-dependent changes in the respiratory cycle, external odor-induced burst discharges occur in TCs mainly during inhalation, while burst discharges of MCs take place during the subsequent exhalation. Thus, we propose that one cycle of respiration correlates to the olfactory perceptual moment (Craig, 2015), i.e., the minimal time duration required for olfactory perception of the external object. This is in agreement with the report of temporal structure of odors to extract the external information (Ackels et al., 2021) and also with that of relation to the respiration cycle (Cury and Uchida, 2010). Individual respiratory cycles may provide a variable quantal time-unit for the olfactory circuitry to transform the odor inputs to behavioral outputs.

#### Timing of Learned Behavioral Decisions in the Olfactory Circuitry

When in the respiratory cycle, does the brain make orthonasal odor-based behavioral decisions? The amygdala plays a key role in establishing the memory of sensory input - reward/punishment association (Janak and Tye, 2015). The CoA is a good candidate area for forming the odor input - reward/punishment association memory. As already mentioned, the orthonasal/external odor information is processed by the multi-synaptic TC pathway during inhalation. Thus, the learned valence signals appear to be generated earlier, presumably in the early exhalation phase or the transition phase between inhalation and exhalation. In contrast, the innate valence signals seem to be generated later in the exhalation phase, because the valence signals transmitted through the direct MC pathway arrive at the CoA during exhalation or in the early exhalation phase. Differential timings of learned and innate valence signals suggest that the learned decision precedes the innate decision.

The MC-mediated innate-valence signals and the TC-mediated learned-valence signals may also converge in the olfactory tubercle, another key area involved in odor-induced motivated behaviors (Ikemoto, 2007; Murata et al., 2015; Wright and Wesson, 2021). It is yet to be studied, however, how the CoA and olfactory tubercle balances the learned and innate decisions to elicit the final behavioral outputs.

### Olfactory Circuitry for Interoception and Exteroception

Olfaction is tightly coupled with respiratory cycles during feeding. OSNs in the nasal cavity receive the orthonasal signals of environmental odors during inhalation, whereas they detect retronasal odor signals of food being chewed during exhalation. The brain receives odor information from the external world via inhalation-phased orthonasal inputs, whereas it detects food-odor information from the oral cavity via exhalation-coupled retronasal inputs (Shepherd, 2006). In contrast to the orthonasal environmental signals during inhalation, it is yet to be studied whether the retronasal internal information in the exhalation phase is transmitted sequentially by the TC and MC pathways.

#### Retronasal Olfaction for Interoceptive Signaling During Eating

Eating is a goal-directed behavior that is critical for the survival of animals. Naturally, the activity of searching and discovering food precedes eating. During food searching, the brain attends to exteroceptive information such as visual and somatosensory cues. Orthonasal odor information is also exteroceptive. It is needed for finding food and also for deciding whether it can be taken into the oral cavity. During the food-consumption behavior, however, the brain switches its attention to interoceptive information such as taste and exhalation-coupled retronasal information. The taste and retronasal olfactory signals within the mouth are parts of interoceptive information of the physiological state in the oral cavity (Craig, 2003; Blankenship et al., 2019). These signals are critical to make a decision whether the food should be swallowed or spit out.

At the end of eating, exhalation-phased retronasal signals and visceral signals from the digestive tract play roles as interoceptive information in evaluating the swallowed food (Shepherd, 2012). Even after eating, food odors released from the blood vessels into the alveolus of the lung are detected by retronasal olfaction as interoceptive signals (Kikuta et al., 2016). Thus, the brain attends to the orthonasal/exteroceptive information during food searching behavior, whereas it switches to the retronasal/interoceptive information of the eating and postprandial scenes.

#### Mirror-Symmetrical Lateral and Medial Maps in the OB

**FIGURE 2:**
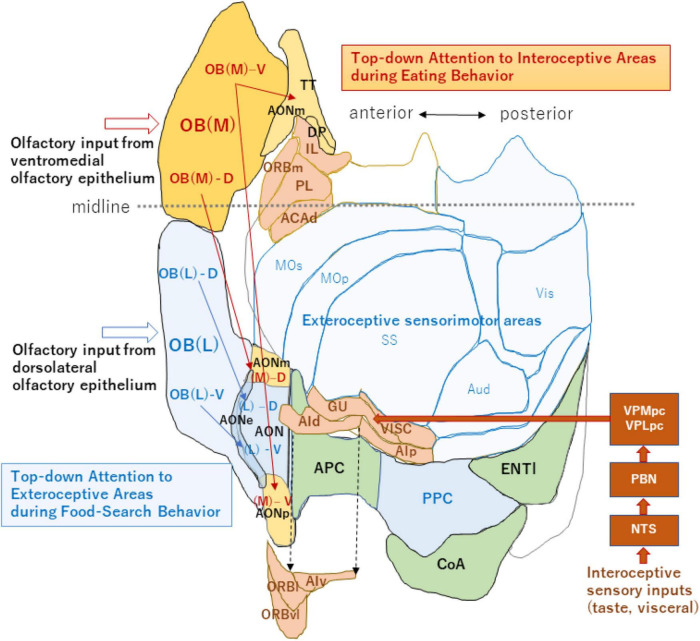
An unfolded map of the neocortex and olfactory cortex (left hemisphere). A dorso-centered view is schematically shown for the exteroceptive and interoceptive sensorimotor regions. Neocortex areas are classified into the exteroceptive sensorimotor (blue) and interoceptive sensorimotor (peach) regions. The exteroceptive region includes the somatosensory (SS), primary motor (MOp), secondary motor (MOs), auditory (Aud), and visual (Vis) areas. The interoceptive region includes the gustatory (GU), visceral (VISC), agranular insular (dorsal part, AId; ventral part, AIv; and posterior part, AIp), orbital (lateral part, ORBl; ventrolateral part, ORBvl, and medial part, ORBm), infralimbic (IL), prelimbic (PL), and dorsal part of the anterior cingulate (ACAd) areas. Interoceptive sensory inputs, including taste and visceral signals reach the interoceptive sensory cortex (including GU and VISC) via the nucleus of solitary tract (NTS), parabrachial nucleus (PBN) and parvocellular parts of the ventral posteromedial thalamic nucleus (VPMpc), and ventral posterolateral thalamic nucleus (VPLpc). AIv, ORBl, and ORBvl are displaced as indicated by the arrows of broken lines to avoid overlap with the olfactory cortex. The medial map of the OB (dark orange), their tufted-cell target areas in the AON and TT (pale orange), and neocortex interoceptive areas (peach) receive the top-down attention signal during eating behavior. The lateral map of the OB (blue), their TC target areas in the AON (blue), and PPC/neocortex exteroceptive areas (pale blue) receive the top-down attention signal during food-searching behavior. This unfolded map was generated using the Allen Institute for Brain Science (2004) (https://mouse.brain-map.org/static/atlas). The midline is shown by a broken line (anterior side is left). For additional abbreviations, please see the legend to Figure 1.

It is well-established that OSNs expressing the same OR species converge their axons to a specific pair of glomeruli in the lateral and medial sides of the OB (Mombaerts et al., 1996). Thus, the two homologous glomeruli are arranged mirror-symmetrically in each OB; one is in the lateral map located in the dorsolateral part of the OB and the other is in the medial map in the ventromedial part (Figure 2; Nagao et al., 2000; Mori and Sakano, 2011). Axon collaterals of TCs connect the two homologous glomeruli via inhibitory interneurons and thus, topographically link the lateral and medial maps (Lodovichi et al., 2003; Marks et al., 2006). OSNs in the dorsolateral OE project their axons to the glomeruli in the lateral map, while those in the ventromedial OE project to the medial map (Saucier and Astic, 1986; Clancy et al., 1994).

It has long been puzzling why there are two redundant maps in each OB. Are the lateral and medial maps functionally the same, or do they possess separate functions? In the neocortex, sensorimotor areas processing the exteroceptive information that guides the somato-motor activity are spatially segregated from those processing the interoceptive information that controls autonomic function (Figure 2; Craig, 2003; Evrard, 2019). Analogous to the functional differentiation between the exteroceptive and interoceptive areas in the neocortex, the lateral and medial maps in the OB may be in charge of processing orthonasal/exteroceptive and retronasal/interoceptive signals, respectively. The ventromedial OE projecting to the medial map in the OB is located closer to the retronasal air flow than the dorsolateral OE projecting to the lateral map. Therefore, the medial map may receive the attention signals from the higher cognitive center as well as from the neuromodulatory system to facilitate the processing of retronasal/interoceptive information during food consumption (Figure 2). In contrast, the lateral map may receive the attention signals to enhance the processing of orthonasal/exteroceptive information during the object-search behavior.

Imaging studies in rodents demonstrate that individual glomeruli in the OB respond to both orthonasal and retronasal stimulation (Gautam and Verhagen, 2012; Sanganahalli et al., 2020). Comparison of orthonasal responses of homologous glomeruli in the lateral and medial maps indicates that these glomeruli exhibit similar activity patterns of response onset latency, rise and decay time, and amplitudes. Interestingly, however, postsynaptic responses in the medial glomeruli demonstrate significantly larger respiration-locked fluctuations than those in the lateral glomeruli. This observation shows that the medial-map circuitry may enhance the respiration-locked activity (Sato et al., 2020). To address this question in terms of exteroception and interoception, direct comparison of glomerular responses is needed between the lateral and medial maps for the orthonasal and retronasal inputs. Unilateral naris-occlusion selectively blocking either inhalation or exhalation may also be of help to clarify the issue.

#### Interoceptive and Exteroceptive Areas in the Olfactory Cortex

Olfaction has two distinct modalities; one is retronasal and the other is orthonasal inputs, although both activate the same set of OR species (Rozin, 1982; Hannum et al., 2018). This observation suggests that not only the OB but also the OC contains two distinct circuitries; one for orthonasal/exteroceptive information and the other for retronasal/interoceptive information. In support of this idea, orthonasal and retronasal inputs are transmitted to the different brain regions (Small et al., 2005; Bender et al., 2009; Chapuis et al., 2009).

Optogenetic inhibition experiments revealed that the insular cortex is needed for the retronasal preference task that requires the memory of retronasal odor - reward association. In contrast, the insular cortex is not needed for the orthonasal preference task that requires the memory of orthonasal odor - reward association, despite that the same odor is used for both the orthonasal and retronasal stimulations (Blankenship et al., 2019). These results indicate that the orthonasal olfactory circuitry is independent of the retronasal one. Furthermore, the memory-engram circuitry of retronasal odor - reward association may be different from that of orthonasal odor - reward association.

Axonal projection of TCs in the medial map is rich in the medial area of the olfactory peduncle including the medial and posterior parts of the AON (AONm and AONp) and tenia tecta (TT) (Figure 2, magenta arrows). Thus, we speculate that if the medial map is specialized for processing interoceptive odor information, the medial area of the olfactory peduncle may also be involved in processing interoceptive signals. In agreement with this hypothesis, a majority of neurons in the ventral TT shows either increased or decreased discharges during eating behavior in which the mouse attends to interoceptive information such as taste and retronasal olfaction (Shiotani et al., 2020).

Because TCs in the lateral OB project their axons mainly to the lateral olfactory peduncle (Figure 2, blue arrows), the lateral area may be for exteroceptive information. It has been reported that neurons in the lateral AON project to the piriform cortex and the posterior piriform cortex forms a learned map of external odor - place association (Poo et al., 2022). Thus, the posterior piriform cortex may be an exteroceptive area responsible for guiding spatial navigation with external odor cues. Further experiments are needed to examine whether each OC area is specialized for processing either the interoceptive or exteroceptive odor signals. Differential delineation of the OB and OC regions into the orthonasal/exteroceptive and retronasal/interoceptive areas will facilitate our understanding of the functional roles of olfactory circuitry in odor-guided motivated behavior.

## Discussion

In this review article, we summarized the recent progress in the study of olfactory circuitry and processing of odor information during respiration. In mammals, odor signals are perceived by OSNs in the OE and converted to a combinatorial pattern of activated glomeruli in the OB. This map information is then transmitted to the AON by TCs for odor recognition and behavioral decisions based on memory (Mori and Sakano, 2021). Input odor signals recall the olfactory scene, further linking it to the valence regions in the amygdala to elicit learned responses. For innate decision, odor information is directly conveyed by MCs from particular functional domains in the OB to specific amygdala nuclei. For example, aversive signals are transmitted from the dorsal OB to the pmCoA (Miyamichi et al., 2011; Root et al., 2014), while attractive social signals are transmitted from the posteroventral OB to the aMeA (Inokuchi et al., 2017). Hard-wired innate behavioral decisions can be modified by imprinted olfactory memory formed during the critical period in neonates (Inoue et al., 2021).

It has been reported that olfactory decisions are made in relation to the respiratory cycle (Kepecs et al., 2006; Mori and Manabe, 2014). The brain receives environmental odor signals only during inhalation and is isolated from the external world during exhalation. External and middle TCs sending odor information to the AON are activated during inhalation, whereas MCs sending signals to the amygdala exhibit burst firing during exhalation. Thus, orthonasal odor signals are transmitted sequentially to the amygdala, first by the multi-synaptic TC pathway during inhalation and then by the direct MC pathway during subsequent exhalation. We assume that retronasal odor signals are also transmitted differentially to the OC and amygdala, first by the TC pathway during early exhalation and then by the MC pathway during late exhalation, although this needs to be further verified by future experiments.

The central olfactory circuitry is designed to perform sequential transmission of odor signals, first by the multi-synaptic learned pathway and then by the direct innate pathway, to assure that the learned decision precedes the innate decision. Since the cooperation of experience-dependent learned decision with the hard-wired innate decision generates better and faster performance in odor-guided tasks than the innate decision alone, precedence of the learned decision appears to be reasonable. Thus, the central olfactory circuitry provides us with an excellent model system for the study of differential cortical processing of sensory inputs for innate and learned decisions.

Respiratory cycles also correlate with food-searching and eating behaviors. We assume that during food-searching and object-detecting behaviors, the brain attends to the exteroceptive lateral OB and the exteroceptive OC areas to facilitate the processing of inhalation-phased orthonasal information. Orthonasal/external odors activate OSNs during inhalation and the OSN activity subsides during the subsequent exhalation. During the inhalation of external odor, the brain detects the increased OSN activity and interprets it as the appearance of external odor. During the subsequent exhalation, the brain detects the diminished OSN activity but does not interpret it as the disappearance of the odor. The exteroceptive areas of the central olfactory circuitry may detect inhalation timing via corollary discharges of respiratory-center neurons in the brainstem (Richter and Smith, 2014). It is possible that the exteroceptive areas differentially perceive the OSN activities in inhalation and subsequent exhalation. During inhalation, a subset of pre-Bötzinger neurons in the respiratory center activates the noradrenergic neurons in the locus coeruleus projecting their axons to the OB and OC (Yackle et al., 2017). Therefore, the activity of pre-Bötzinger neurons during inhalation may be transmitted to the exteroceptive area, so that the processing of inhalation-phased orthonasal information can be facilitated during food-searching and object-detecting behaviors.

We also speculate that during food-consumption, the brain attends to the interoceptive medial map in the OB as well as to the interoceptive area in the OC to facilitate the processing of exhalation-phased retronasal information. Retronasal/internal odors activate OSNs during exhalation and the OSN activity may subside during the subsequent inhalation. During the exhalation, the brain detects the increased OSN activity and interprets it as the appearance of internal odor. During the subsequent inhalation, the brain detects the diminished OSN activity but does not interpret it as the disappearance of the internal odor. We assume that the interoceptive area in the olfactory circuitry may detect exhalation timing and differentially perceive the OSN activity during exhalation as well as subsequent inhalation. Agranular insular areas (AIv and AIp) and the lateral orbitofrontal area (OBRl) receive the inputs not only from the OC but also from the neocortical interoceptive areas such as the gustatory area (Rolls, 2019; Mizoguchi et al., 2020). Therefore, we speculate that during the behavior of food-consumption, these areas may integrate the neocortical interoceptive activity with the retronasal/interoceptive activity, which is relayed by the medial OB map and the interoceptive OC area, to generate the perception of food flavor (Shepherd, 2006; Small, 2012).

How does the orthonasal activity in the exteroceptive areas of the OB and OC interact with the retronasal odor-induced activity in the interoceptive areas? This is an important issue to be studied in the future for our understanding of not only the olfactory system but also the whole brain. The mouse olfactory system will continue to serve as an excellent model system for the study of sensory processing in relation to the respiratory cycle in mammals.

## Author Contributions

Both authors contributed equally to this work.

## Conflict of Interest

The authors declare that the research was conducted in the absence of any commercial or financial relationships that could be construed as a potential conflict of interest.

## Publisher’s Note

All claims expressed in this article are solely those of the authors and do not necessarily represent those of their affiliated organizations, or those of the publisher, the editors and the reviewers. Any product that may be evaluated in this article, or claim that may be made by its manufacturer, is not guaranteed or endorsed by the publisher.
